# Asymptomatic Severe Hypocalcemia Secondary to Vitamin D Deficiency in an Elderly Patient

**DOI:** 10.1155/2011/830952

**Published:** 2011-11-02

**Authors:** Saleh Aldasouqi, Crystal M. Glassy, Matthew S. Glassy, Anxhela Treska, Molly Caldwell-McMillan, Ved Gossain

**Affiliations:** ^1^Endocrine Division, Department of Medicine, Michigan State University, Lansing, MI 48910, USA; ^2^College of Osteopathic Medicine, Michigan State University, Lansing, MI 48910, USA; ^3^College of Human Medicine, Michigan State University, Lansing, MI 48910, USA; ^4^Georgia Health Sciences University, Augusta, GA 30912, USA; ^5^Washington University School of Medicine in St. Louis, St. Louis, MO 63110, USA

## Abstract

*Objective*. To describe an asymptomatic presentation of severe hypocalcemia secondary to vitamin D deficiency in an elderly patient. *Methods*. We describe the presentation and clinical course of an elderly woman with asymptomatic severe hypocalcemia referred to an endocrinology clinic for hyperparathyroidism. *Results*. The patient is an 83-year-old Caucasian woman who presented to an endocrinology clinic for evaluation of hyperparathyroidism, with an intact PTH of 462 pg/mL (normal range 14–72 pg/mL). The same lab report included a serum calcium of 5.2 mg/dL (normal range 8–10.5 mg/dL). She displayed no signs or symptoms of hypocalcemia. Given the extreme severity of hypocalcemia and her age, she was hospitalized. Vitamin D deficiency was suspected and was subsequently confirmed with undetectable serum levels. The patient remained asymptomatic throughout her hospital stay. Total and ionized calcium levels at discharge were 7.2 mg/dL and 1.03 mmol/L (normal range 1.1–1.4 mmol/L), respectively. *Conclusion*. Physicians should exercise prudent management with respect to the vitamin D status of the elderly patient, as certain patients may exhibit severe hypovitaminosis D and hypocalcemia without apparent clinical symptoms.

## 1. Introduction

Vitamin D deficiency is particularly important within the elderly population due to its direct relationship with increased morbidity and frailty [1]. Vitamin D deficiency is common in the elderly [2, 3]. The prevalence of vitamin D deficiency ranges from 5–25% in independent, community dwelling elderly, to 48–80% in institutionalized elderly [4–6]. Vitamin D deficiency is an established risk factor for osteoporosis, falls, and fractures within the elderly. Additionally, vitamin D deficiency is associated with breast, prostate and colon cancer, type 2 diabetes, and cardiovascular disorders such as hypertension [7], conditions which commonly afflict the elderly.

Due to vitamin D's integral role in the absorption of calcium, more severe vitamin D deficiency may result in hypocalcemia that may vary in clinical manifestations. In general, hypocalcemia has a spectrum of clinical manifestations. In mild or chronic hypocalcemia, symptoms may be few, such as numbness or muscle cramps, or they may be absent. In severe or acute hypocalcemia, symptoms may be life threatening. Presentations may include severe neuromuscular irritability, tetany, papilledema, arrhythmias, seizures, or death [8, 9].

Vitamin D deficiency as a cause of hypocalcemia, though well documented in the literature, has received little attention, and only a few reports have scrutinized its etiologic role, as reported by Noto and Heller [10]. A case of extremely severe hypocalcemia (5.5 mg/dL) has been reported due to vitamin D deficiency [11], but this was in a 69-year-old patient with prior bariatric surgery, and this patient was symptomatic. Our patient's calcium was lower, at 5.2 mg/dL, and was asymptomatic. We are not aware of reported cases of extremely severe hypocalcemia due to nutritional vitamin D deficiency, in an ambulatory elderly individual, who was asymptomatic.

We hereby present a case of an elderly women with severe hypocalcemia and hypovitaminosis D without apparent clinical symptoms. The patient was eucalcemic eight months prior to presentation.

## 2. Presentation of the Case

An 83-year-old married Caucasian female presented to an endocrinology clinic in Lansing, Michigan for evaluation of hyperparathyroidism in March of 2005. A few days prior to her visit, the patient had labs drawn which had shown an iPTH of 462 pg/mL and total calcium of 5.2 mg/dL. Eight months prior, her calcium was 8.5 mg/dL. She lived all her life in a small town in Mid-Michigan, USA (a region with long winters and insufficient clear sunny skies). Despite the severity of her current hypocalcemia, there was a discordant absence of hypocalcemic symptoms. She did admit to occasional numbness and spasms of the hands one week prior to presentation which had since spontaneously resolved. The patient denied muscle weakness or aches, carpopedal spasms, paresthesias, or seizures. The patient admitted to poor dietary intake of milk and dairy products, as well as little sunlight exposure. A complete review of systems was negative aside from weight loss. 

The patient's medical history was significant for hypothyroidism after partial thyroidectomy 40 years prior for which she was taking 75 mcg of L-thyroxine daily. An other medical history included dementia for which she received 5 mg of Aricept daily. She did not report the use of any herbals or nonprescription medications, and denied smoking, alcohol, and illicit drug use. Her family history was noncontributory. 

At this time, vitamin D deficiency was suspected as a cause for her hypocalcemia. Given the severity of hypocalcemia and advanced age, she was directly admitted to the hospital. 

On admission to the hospital, the patient appeared comfortable. Her blood pressure was 150/78, pulse 74, respirations of 16, and temperature of 98.1. Her neurological examine revealed mild cognitive impairment. Trousseau's and Chvostek's signs were negative. Cardiac exam revealed a 3/6 systolic murmur. The remainder of the examination was within normal limits. Admission laboratory studies are summarized in Table 1, and admission EKG showed a regular sinus rhythm with no QT prolongation. 

Repletion of calcium was undertaken with both IV and oral calcium. She was additionally started on vitamin D supplementation. During the course of her 5-day hospital stay, the patient remained asymptomatic. The patient was given oral and IV calcium and upon discharge her total calcium was 7.2 mg/dL and ionized calcium 1.03 mmol/L. 

Discharge medications included donepezil 5 mg p.o. daily, magnesium oxide 400 mg p.o. twice daily, levothyroxine sodium 50 mcg p.o. daily, calcitriol 0.25 mcg p.o. daily, calcium carbonate 1000 mg p.o. three times daily, and hydrochlorathiazide 12.5 mg p.o. daily. She was advised to followup with the endocrinology clinic in 2 weeks. At subsequent outpatient followup, her calcium was maintained between 8 and 9 mg/dL.

## 3. Discussion

The etiologies of vitamin D deficiency in the general population are multifactorial and well established in the literature. Nutritional and environmental factors are among the commonest etiologies. The elderly are at a higher risk for developing vitamin D deficiency. Age-related changes resulting in decreased production of vitamin D by the skin, progressive loss of renal function, and a naturally occurring decrease in appetite; all contribute to this risk. Our patient exhibited additional risk factors such as living in a northern locale far from the equator, having limited exposure to sunlight, and a self-reported diet low in vitamin D. 

Higher 25-hydroxyvitamin D status correlates with better bone and muscle health. A meta-analysis in 2004 showed that vitamin D reduced the risk of falling by 22% in older adults [1]. Similar findings were observed in subsequent epidemiological studies [1]. Calcium adequacy is also critical for fracture prevention. Low calcium levels lead to weak bone susceptible to fracture. Considering that one out of every three older adults falls each year, hypovitaminosis D can result in significant morbidity and mortality among the elderly [12].

The active form of vitamin D, indirectly and directly regulates hundreds of genes, including blunting renin production in the kidney [13]. Several epidemiological studies have shown a relationship between elevated systolic blood pressure and vitamin D deficiency [13]. It is worthwhile to note that a cohort of Framingham patients with vitamin D deficiency and HTN has exhibited a higher CV risk of fatal or nonfatal MI, ischemia, stroke, or heart failure [13].

Additionally, secondary hyperparathyroidism has also been associated with a doubling of CV disease and mortality [13]. It is reasonable then to hypothesize that our asymptomatic patient with vitamin D deficiency, secondary hyperparathyroidism, and HTN may have been at very high risk for CV complications. However, given her asymptomatic presentation, her very high risk status could have remained unknown.

However, of all the ramifications of the role of vitamin D in health and disease and of all the health consequences of vitamin D deficiency, the issue of hypocalcemia has received little attention in the literature [10]. The etiology of severe hypocalcemia in our patient was clearly the severe degree of vitamin D deficiency, with 25-OH vitamin D being extremely low (almost undetectable). This deficiency appears to be purely environmental and nutritional in origin. Her kidney function was perhaps appropriate for her advanced age, and even if some degree of renal insufficiency could be arguably made as a contributing etiology, the renal activation of vitamin D would be less relevant due the extreme deficiency of the substrate. We opted to treat our patient with calcitriol to expedite her metabolic recovery. Certainly, high doses of vitamin D2 or D3 would be a reasonable alternative.

Our patient was ambulating with a severe state of hypocalcemia, and, at her advanced age, she was at heightened risk of serious, and potentially life-threatening complications should this routine lab order have not been ordered. We suggest that physicians should be extremely cognizant of elderly patient's vitamin D and calcium status even if they do not present with symptoms typical of hypovitaminosis D and/or hypocalcemia.

## 4. Conclusion

In summary, physicians should exercise prudent management with respect to vitamin D and calcium status in the elderly patient. Certain patients may exhibit severe hypovitaminosis D and hypocalcemia without apparent clinical symptoms, as did our patient.

## Figures and Tables

**Table 1:** Initial blood chemistry studies.

Test (unit)	Value (normal range)
Serum calcium (mg/dL)	5.2 (8–10.5)
Ionized calcium (mmol/L)	0.79 (1.1–1.4)
BUN (mg/dL)	16 (7–18)
Serum creatinine (mg/dL)	1.4 (0.5–1.4)
Phosphorous (mg/dL)	4.7 (3.0–4.5)
Albumin (mg/dL)	3.4 (3.5–4.8)
Alkaline phosphatase (U/L)	313 (20–70)
Serum iPTH (pg/dL)	462 (14–72)
25-hydroxyvitamin D (pg/dL)	4 (10–55)

Blood urea nitrogen (BUN); intact parathyroid hormone (iPTH).
